# Supplementary material for: Complex‐centric proteome profiling by SEC‐SWATH‐MS
Source: Mol Syst Biol. 2019 Jan 14;15(1):e8438. doi: 10.15252/msb.20188438 (PMC6346213; doi:10.15252/msb.20188438)
Supplement: Supplementary file 8 — Dataset EV7 [file MSB-15-e8438-s008.zip › feature_plots_string/P63244.pdf]

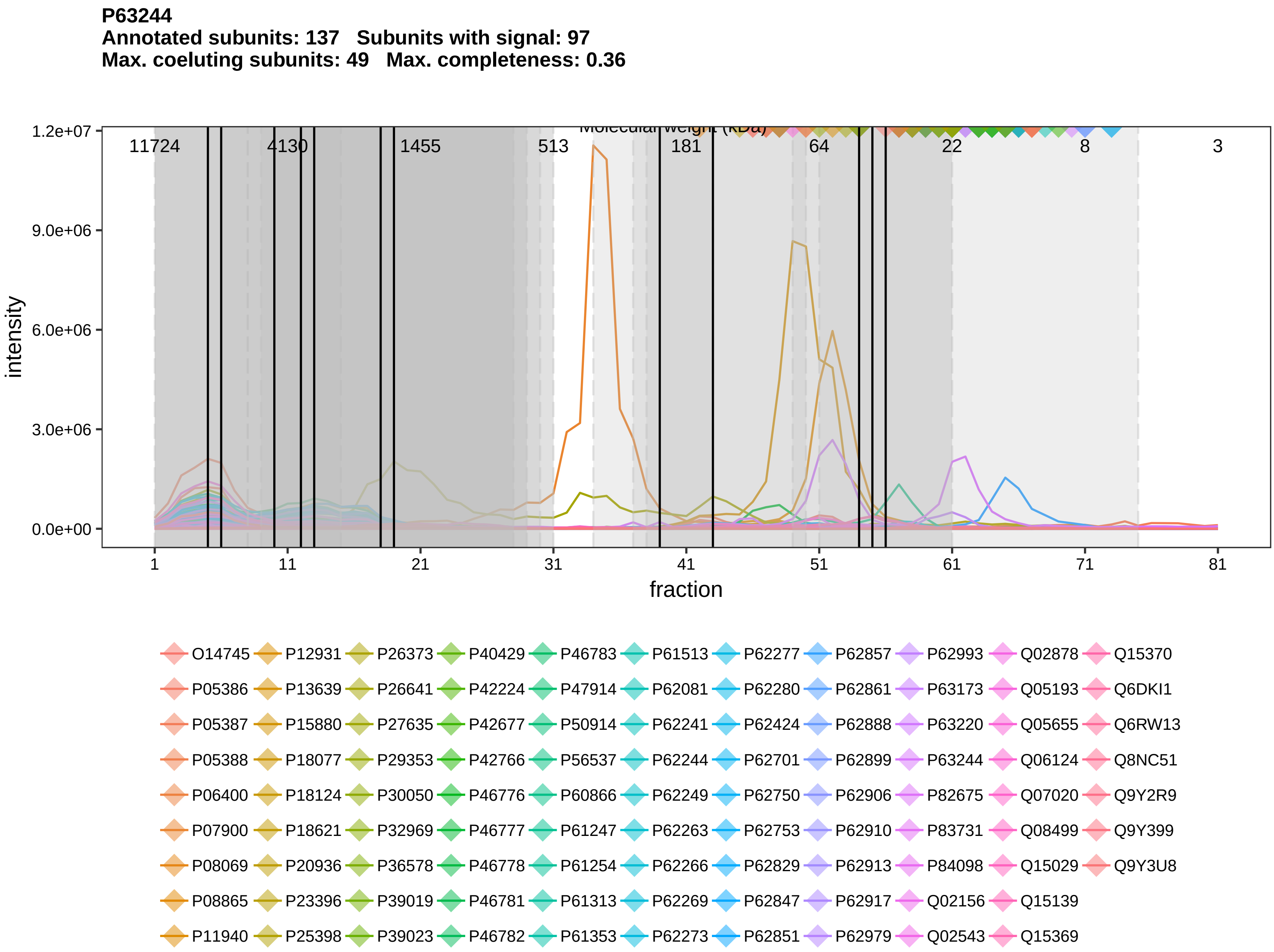
P63244
Annotated subunits: 137 Subunits with signal: 97
Max. coeluting subunits: 49 Max. completeness: 0.36
1.2e+07
9.0e+06
6.0e+06
3.0e+06
0.0e+00
intensity
11724
4130
1455
513
181
64
22
8
3
1
11
21
31
41
51
61
71
81
fraction
O14745
P05386
P05387
P05388
P06400
P07900
P08069
P08865
P11940
P12931
P13639
P15880
P18077
P18124
P18621
P20936
P23396
P25398
P26373
P26641
P27635
P29353
P30050
P32969
P36578
P39019
P39023
P40429
P42224
P42677
P42766
P46776
P46777
P46778
P46781
P46782
P46783
P47914
P50914
P56537
P60866
P61247
P61254
P61313
P61353
P61513
P62081
P62241
P62244
P62249
P62263
P62266
P62269
P62273
P62277
P62280
P62424
P62701
P62750
P62753
P62829
P62847
P62851
P62857
P62861
P62888
P62899
P62906
P62910
P62913
P62917
P62979
P62993
P63173
P63220
P63244
P82675
P83731
P84098
Q02156
Q02543
Q02878
Q05193
Q05655
Q06124
Q07020
Q08499
Q15029
Q15139
Q15369
Q15370
Q6DKI1
Q6RW13
Q8NC51
Q9Y2R9
Q9Y399
Q9Y3U8
